# Comparative cell cycle transcriptomics reveals synchronization of developmental transcription factor networks in cancer cells

**DOI:** 10.1371/journal.pone.0188772

**Published:** 2017-12-11

**Authors:** Johan Boström, Zuzana Sramkova, Alena Salašová, Helena Johard, Diana Mahdessian, Radek Fedr, Carolyn Marks, Jiřina Medalová, Karel Souček, Emma Lundberg, Sten Linnarsson, Vítězslav Bryja, Petra Sekyrova, Mikael Altun, Michael Andäng

**Affiliations:** 1 Science for Life Laboratory, Division of Translational Medicine and Chemical Biology, Department of Medical Biochemistry and Biophysics, Karolinska Institutet, Stockholm, Sweden; 2 Department of Physiology and Pharmacology, Karolinska Institutet, Stockholm, Sweden; 3 Central European Institute of Technology, Masaryk University, Brno, Czech Republic; 4 Department of Medical Biochemistry and Biophysics, Karolinska Institutet, Stockholm, Sweden; 5 Science for Life Laboratory, KTH—Royal Institute of Technology, Stockholm, Sweden; 6 Department of Cytokinetics, Institute of Biophysics CAS, v.v.i., Královopolská 135, Brno, Czech Republic; 7 International Clinical Research Center, Center for Biomolecular and Cellular Engineering, St. Anne’s University Hospital in Brno, Brno, Czech Republic; 8 Department of Experimental Biology, Faculty of Science, Masaryk University, Brno, Czech Republic; Virginia Commonwealth University, UNITED STATES

## Abstract

The cell cycle coordinates core functions such as replication and cell division. However, cell-cycle-regulated transcription in the control of non-core functions, such as cell identity maintenance through specific transcription factors (TFs) and signalling pathways remains unclear. Here, we provide a resource consisting of mapped transcriptomes in unsynchronized HeLa and U2OS cancer cells sorted for cell cycle phase by Fucci reporter expression. We developed a novel algorithm for data analysis that enables efficient visualization and data comparisons and identified cell cycle synchronization of Notch signalling and TFs associated with development. Furthermore, the cell cycle synchronizes with the circadian clock, providing a possible link between developmental transcriptional networks and the cell cycle. In conclusion we find that cell cycle synchronized transcriptional patterns are temporally compartmentalized and more complex than previously anticipated, involving genes, which control cell identity and development.

## Introduction

The cell cycle coordinates a series of changes that result in the initiation of specific core functions at different cell cycle stages, supporting, for example, DNA replication, quality control and cell division. One level of control in this process is exercised by feed-forward and feedback loops of posttranslational modifications and protein degradation. Another level of control is maintained via regulated transcription. The transcriptional changes that occur during the cell cycle in mammalian cells are associated with cell cycle transition points: G1-to-S, G2-to-M and M-to-G1[1]. By far, the most well studied point of transcriptional control is the G1-to-S transition, where S phase transcription is activated by E2F1-3, members of the E2F family of transcription factors (TFs), after they are released from the hyperphosphorylated RB protein [1–3] and Cyclin/CDK complexes. This activation is followed by transcriptional repression later in the S phase by E2F4-8 and pocket proteins p107 and p130 in late S phase [1, 4].

In addition to controlling basic functions such as replication and cell division, the cell cycle also affects the maintenance of and changes in cell identity and specification during development. For example, in human embryonic stem cells, Cyclin Ds control TGF-β signalling and the activation status of SMAD2/3, rendering the cell susceptible to differentiation along different lineages in early versus late G1 phase [5]. Likewise, in neural progenitor cells, Notch signalling integrates with the cell cycle to impact neural lineage specification [6, 7]. Further adding to this complex view of the cell cycle, WNT/Frizzled/β-catenin signalling shows a peak in G2 in some cells [8, 9]. TFs that oscillate during the cell cycle may interact with each other in transcriptional networks that can facilitate a series of successive activation or repression steps [1, 10, 11]. This might result in a domino-like effect, feeding information forward through the cell cycle, potentially with some degree of independence. Indeed, studies in yeast cells have shown that up to 20% of the TFs that oscillate with the cell cycle are non-essential TFs [12], and these genes may become self-sustained in their oscillatory behaviour in the absence of the cyclin/CDK-based cell cycle [13]. Such TF networks may thus constitute semi-autonomous transcriptional networks that propagate information through the cell cycle. Adding another layer of control, the circadian molecular clock is an independent transcriptional oscillator that reciprocally synchronizes with the cell cycle [14–17].

To map both core and cell-type-specific cell cycle-dependent transcription, we examined the HeLa cell cycle transcriptome and compared it to that of U2OS cells using RNA sequencing of cells sorted according to cell cycle without chemical synchronization. To efficiently map cell cycle oscillators and categorize distinct cell cycle phase patterns, we developed a novel algorithm, TriComp. This algorithm calculates two comprehensible variables that describe complex relationships between three quantitative variables, i.e., levels of expression in three cell cycle phases. Moreover, it allows for straightforward comparisons between different types of data sets. Patterns identified by the TriComp algorithm were superimposed onto established TF networks to reveal the potential temporal succession of gene expression, reflecting activation/repression hierarchies involved in cell cycle oscillation.

## Results

### Fucci-based separation of cell cycle phases and deep RNA sequencing

**Fig 1:**
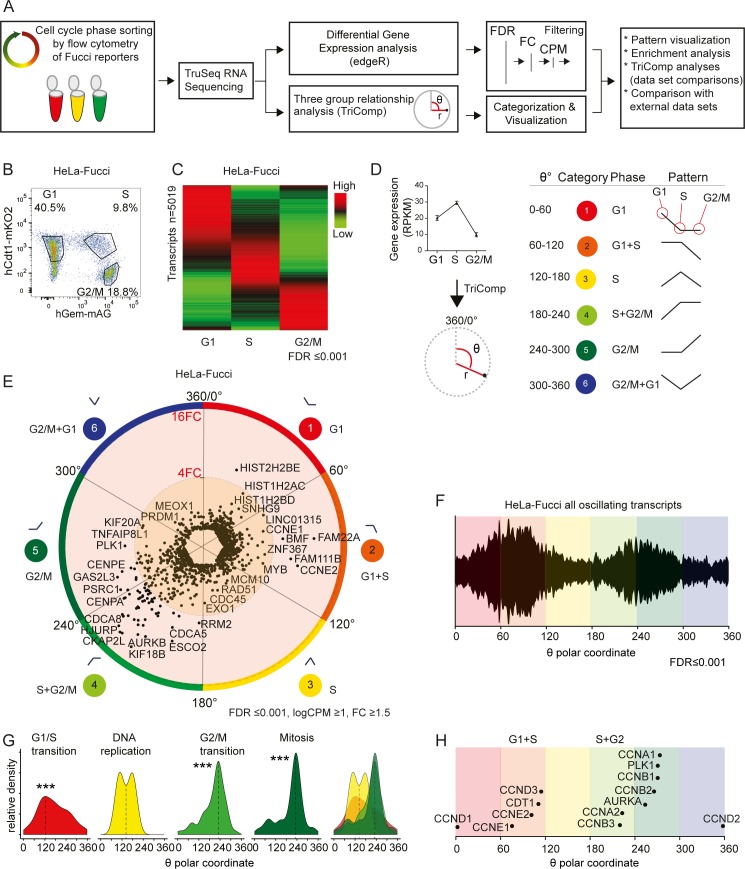
Visualization of transcripts oscillating with the cell cycle reveals expression patterns. (A) The scheme of the experimental and analytical workflow. (B) HeLa-Fucci cells were sorted into three restricted cell cycle phases, G1, S, and G2/M, according to the expression of Fucci markers. (C) A heat map analysis shows differential gene expression patterns in sorted HeLa-Fucci cells. (D) For each individual transcript in the HeLa-Fucci transcriptome, the TriComp algorithm was used to calculate polar coordinates (θ, *r*). θ denotes the relationship between gene expression levels in the three cell cycle phases, and r denotes the intensity of this relationship. Genes with similar expression patterns were divided into six categories (6x60°) based on their θ-value. (E) Polar coordinates for all transcripts found to oscillate significantly in synchrony with the cell cycle (FDR≤0.001, logCPM ≥1, FC ≥1.5) in sorted HeLa-Fucci cells show an unequal distribution. (F-H) θ-values describing the distribution of relationship patterns of oscillation for all significantly oscillating transcripts in HeLa cells (FDR≤0.001) (F), transcripts belonging to GO terms associated with the cell cycle (H), and common cell cycle markers (G). Significance stars denote the probability of arising from the same distribution as the full set of significantly oscillating transcripts using log-likelihood-ratio statistical comparison with a six-group binning (***: p < 0.001).

Previous analyses of cell cycle-dependent transcription have in most cases used chemically synchronized cells [18, 19]. Live cell cycle phase reporters such as the Fucci system [20] provide an alternative method that has been explored in recent analyses of the cell cycle in, for example, embryonic stem cells [5, 21, 22]. To characterize the relationship of the Fucci probes to the mitotic cell cycle, we analyzed chromatin content and DNA replication status and visualized it overlaid on Fucci reporter fluorescence, Panel A in S1 Fig. This experiment establishes that the majority of actively replicating cells are mAG-hGem^+^, mKO2-hCdt1^+^. We then used Fucci reporters to sort live, unsynchronized cells into discrete populations corresponding to non-replicating 1X-chromatin cells (G1-phase), actively replicating cells (S-phase) and non-replicating 2X-chromatin cells (G2/M-phase) by flow cytometry (Fig 1A and 1B, S1 Table). Control samples of sorted populations were fixed and analysed for cell cycle phase based on DNA content, demonstrating the correlation between Fucci-based and DNA content-based cell cycle phase determination (Panels A,B in S2 Fig). Three cell cycle phase populations from two cell lines, HeLa-Fucci and U2OS-Fucci, were analysed for gene expression by RNA sequencing. The data were processed by an ANOVA-like differential gene expression approach from the edgeR package [23]. Stringency cut-offs were used for the calculated false discovery rate (FDR), average gene expression (logCPM) and maximal fold change (FC) for each gene (Panels C,D in S2 Fig, S2 Table). In this report, we focused on transcript levels and disregarded splice variants. However, the information on splice variants is available for reuse in GEO:ID(PLACEHOLDER: To be submitted). Of the transcripts with measurable mRNA levels, approximately one-third significantly oscillated in synchrony with the cell cycle (FDR≤0.001) (Fig 1C, Panel E in S2 Fig), possibly representing known cell cycle regulated genes as well as unidentified genes expressed in a cell cycle-dependent manner.

### A new algorithm for comparisons between three groups of data

To classify and visualize the expression patterns of genes that are regulated over the three sorted cell cycle phases, we developed a novel algorithm for simultaneous comparisons between three groups of data, TriComp. The algorithm generates an angular coordinate for each gene (θ) that reveals the relationship pattern between the three temporal stages and a radial coordinate (r) that reflects the extent of the relationship pattern based on the FC between the highest and lowest expression values in the cell cycle phases (Fig 1D, S2 Table).

Every possible relationship pattern corresponds to a specific θ-value, but the θ-value does not store any information about the intensity of such a relationship (this is stored in the r-value). For example, the relationship between the three expression values (1 | 2 | 4) and (1 | 10 | 100) which both share the relationship (group two being logarithmically exactly halfway between group 1 and 3) share the same θ-value (240°) with the latter having a much higher r-value. The same principle holds true with the two groups of values; (1 | 1 | 1.01), and (1 | 1 | 14682). Both have a θ-value of 300° but widely different r-values. This highlights the importance of including filtering both on statistical significance and biological significance when analyzing data using θ-values. Using these two coordinates reduces the complexity of the three-group comparisons, enables meaningful categorization and provides informative visualization. Moreover, the θ-value (after filtering based on applied statistics and r-value) can be used for comparisons between experiments, between cell lines, and between different data sets.

To categorize genes with similar expression patterns, the spectrum of relationships was divided into 6 categories based on θ-value (Fig 1D, Panel G in S2 Fig). Each θ-category corresponds to a binary classification of gene expression over the three phases relative to each other. Categories 1, 3 and 5 represent patterns of gene expression that peak in a single cell cycle phase: G1, S or G2/M. Categories 2, 4 and 6 represent a composite pattern where gene expression is high in two consecutive cell cycle phases and low in the third phase, i.e., high in G1+S, S+G2/M and G2/M+G1.

The distribution of transcript expression patterns was visualized by projecting the θ and r coordinates of 1132 transcripts in the HeLa-Fucci transcriptome (FDR≤0.001, logCPM>1, FC≥1.5) in a circular graph (Fig 1E). To inspect this distribution more closely, we plotted the θ-values for all significantly oscillating transcripts in HeLa cells (Fig 1F). This uncovered a pattern of genes with high transcript levels in categories 2 (G1+S) and 5 (G2/M) and low transcript levels in categories 3 (S phase) and 6 (G2/M+G1 phase). These results suggest that the expression of a large number of transcripts is controlled throughout the cell cycle.

We validated the TriComp analysis of our sequencing data by correlating the θ-values with Gene Ontology terms associated with the cell cycle (S3 Table). GO terms associated with G1/S transition were found in θ-categories 2 and 3, GO terms involved in DNA replication were restricted to θ-categories 2 and 3, and GO terms related to G2/M transition and mitosis contained genes from θ-categories 5 and 6 (Fig 1G). A visual alignment of θ-values for recognized cell cycle phase markers verified the expected order of expression, in which the transcripts for Cyclin D1/2/3 and Cyclin E1/2 were succeeded by those for Cyclin A1/A2, B1/B2, Aurora kinase A and Polo-like kinase 1 (Fig 1H). These results show that the TriComp algorithm enables straightforward comparisons between the three cell cycle phases and reveals the temporal succession of cell cycle regulated gene expression.

### Validation of TriComp analyses and data comparisons

**Fig 2:**
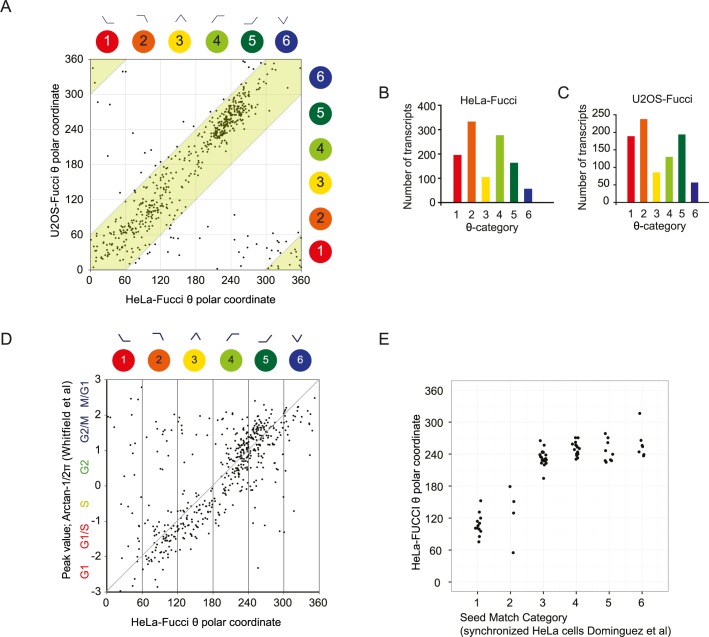
Comparisons of different cell cycle data sets show consistent expression patterns. (A) Comparative analysis of HeLa-Fucci and U2OS-Fucci cell cycle-dependent transcriptomes after plotting their respective θ-value for each shared transcript (stringency level: FDR≤0.001). The green area denotes a correlation of less than 60 degrees in the θ-value. (B, C) Number of transcripts in each of the six TriComp θ-categories (FDR≤0.001). (D) Plot of the θ-value for HeLa-Fucci transcripts versus the reported phase peak value for each probe reported by Whitfield *et al*.[19]. (E) Category distribution plots for the core set of 67 genes reported by [18] versus the θ-value for genes significantly oscillating (FDR≤0.001) in HeLa-Fucci cells.

To further validate the TriComp-based analysis, we compared genes oscillating in a cell cycle-dependent manner shared by the HeLa-Fucci and U2OS-Fucci data sets by plotting the θ-value for all significantly oscillating transcripts. The direct comparison showed a clear correlation between the two data sets, as 90% of the genes had a θ-value difference lower than 60 degrees (Fig 2A). Interestingly, the transcriptional pattern observed in the HeLa-Fucci data set (Fig 1F, Fig 2B) was also observed in the U2OS-Fucci data set (Fig 2C), indicating that regardless of cell type, similar transcriptional regulation occurs in a time-specific manner.

The θ-value derived from the TriComp algorithm can be readily used for comparisons between data sets where the temporal descriptor is of another character. To externally validate our data set, the HeLa-Fucci cell cycle data were compared to two previously published cell cycle data sets [18, 19]. These studies used chemically synchronized HeLa cells and sinusoidal curve fitting and pattern matching from multiple microarray time-point analyses to map the oscillations of the transcriptome. After mapping the Whitfield *et al*. probe results to our transcripts using Gene IDs and BioMart, we compared the peak position of the sinusoidal curve fitting (Arctan2) to the TriComp θ-value π-values (Fig 2D). Out of 723 single-gene unique probes in the Whitfield *et al*. study, 505 probes were identified to oscillate in our data set as well (FDR≤0.001, FC≥1.1.) (Panel A in S3 Fig).

We next compared our data to Dominguez *et al*., and of the 1182 genes identified to oscillate by their study, a total of 1078 genes matched gene names in our data set. 589 of these genes significantly oscillated in our data set (at FDR≤0.001, FC≥1.1). Dominguez *et al*. allocated oscillating genes into 6 categories, and plotting these categories against the TriComp θ-values from our data set showed a similar distribution pattern over the cell cycle (Panel B in S3 Fig). In addition, all of the 67 genes defined by Dominguez et al. as the cell cycle "core set" were found to be statistically significantly oscillating in our data set and the oscillation patterns correspond to our data (Fig 2E).

Together, comparisons to other cell cycle transcriptome experiments showed similar results for transcripts that could be matched in corresponding data sets. Our RNA sequencing identified over 5000 transcripts (including noncoding transcripts) that exhibited cell cycle-dependent oscillatory behaviour (Panel E in S2 Fig), providing a potential opportunity to identify genes with as yet uncovered cell cycle regulatory effects.

### Patterns of TF expression during the cell cycle

**Fig 3:**
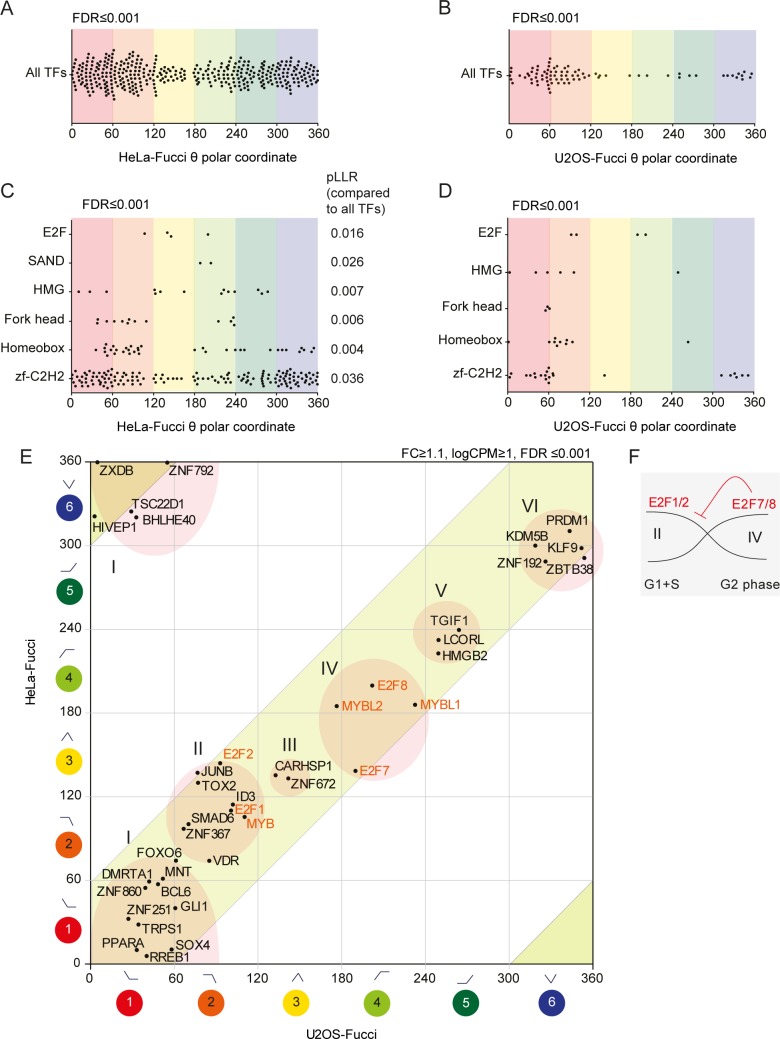
Large TF families oscillate and show differential distribution during the cell cycle. (A-D) θ-value plots for all TFs and TF families with a significantly differing pattern in (A, C) HeLa-Fucci cells and (B, D) U2OS-Fucci cells (FDR ≤0.001; logCPM ≥1; FC ≥1.5). (E) θ-value plot of cell-cycle-synchronized TFs in HeLa-Fucci cells versus U2OS-Fucci cells, with suggested phase-specific expression groups denoted I-VI and the main TFs involved in G1 restriction and onset of S phase indicated in red. (F) Schematic showing how repressor activity may shape transcriptional boundaries during the cell cycle, exemplified by E2F7/8 repression of E2F1/2 expression.

The pattern observed in Fig 1F indicated extensive transcriptional regulation during the cell cycle. Therefore, we examined the expression patterns of TFs, as regulators of transcription, more closely. To search for potential patterns in TF expression correlating with cell cycle progression, we mapped transcripts coding for TFs in both the HeLa and U2OS data sets by filtering the transcriptome data using a list of known TFs (Animal TFDB (Ref: PMID 22080564)), comprising 1591 genes in total. We identified 390 TFs in HeLa cells and 82 TFs in U2OS cells that significantly oscillated (FDR≤0.001) above the 1.1 FC level (S4 Table). Mapping the θ-values of significantly oscillating TFs in HeLa (Fig 3A) and U2OS (Fig 3B) cells showed a pattern similar to that of all significantly oscillating transcripts in Fig 1F. We next subdivided the transcriptome data into discrete TF subfamilies and analysed the distribution of the θ-values in HeLa (Fig 3C) and U2OS (Fig 3D) cells. Interestingly, apart from the core cell cycle machinery that included TFs like E2F family, other TF families with a high number of oscillating genes included developmental patterning and cell identity genes. The E2F TFs clustered in θ-categories spanning the S phase, while developmental TF families such as Homeobox, Forkhead and zf-C2H2 clustered predominantly in θ-categories 1–2 or 5–6, but not in category 3 (S phase). A STRING[24] analysis of the oscillating TFs in HeLa cells (Panel C in S3 Fig) suggested extensive network integration of a large number of TFs, with nodes such as MYC, TP53, NCOR1, PPARA and SMAD3 appearing to link the cell cycle to TFs associated with neurodevelopment (such as PAX6, ISL1, LHX4 and DLX1) and the circadian clock (ARNTL).

**Fig 4:**
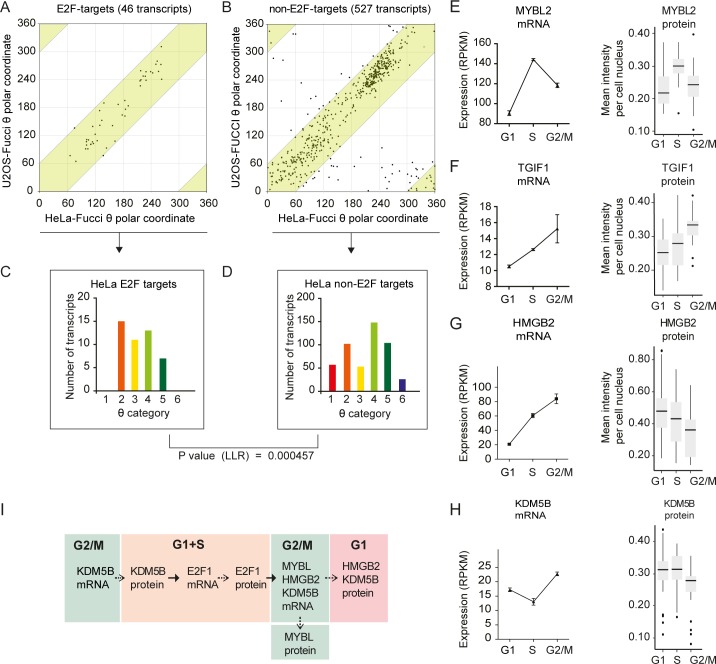
E2F1-controlled gene expression is compartmentalized. (A-D) Comparative analysis of E2F (A, C) and non-E2F targets (B, D) was performed using both the HeLa-Fucci and U2OS-Fucci cell cycle-dependent transcriptomes by plotting θ-values, with expression groups 2–5 highlighted by a pink box (A, B), and a distribution analysis with a log-likelihood ratio statistical comparison using the HeLa-Fucci cell θ-values (C, D). (E-H) mRNA expression in the HeLa-Fucci data set (RPKM), and protein levels as Tukey boxplots of average antibody intensities per cell nucleus, subdivided by cell cycle phase according to the quantification of Fucci reporters using fluorescent imaging. Error bars denote SEM. (I) An example of known temporal relationships among cell cycle-controlled TFs, verified by our data.

To analyse the phase distribution of the TFs shared between HeLa and U2OS cells, respective θ-values were plotted, showing a high degree of correlation (all shared TFs exhibited a θ-value within 75 degrees of each other) (Fig 3E). At the level of FC≥1.1 and logCPM≥1, we found 39 TFs with significantly oscillating expression that were shared in both data sets. According to θ-values, we could identify six consecutive groups, I-VI, throughout the cell cycle (Fig 3E), indicating that transcriptional regulation follows a pattern of temporal succession. Illustrating this idea, the θ-values for the E2F1 and E2F2 transcripts peaked earlier than those for the E2F7 and E2F8 transcripts (the former clustering with group II and the latter in group IV), creating a known activator/repressor loop (Fig 3F) [1]. Further analysis of E2F1 and its known target genes (using a list of 130 E2F targets from Bracken *et al*. 2004) identified 46 transcripts (out of 110 gene names found in our data sets) that significantly oscillated in both the HeLa and U2OS data sets. The distribution of E2F targets and non-E2F targets in HeLa or U2OS cells was visualized by plotting the respective θ-value (Fig 4A and 4B) as well as a number of genes in each θ-category (Fig 4C and 4D). E2F target transcripts were present in TriComp θ-categories 2–5, which span the G1/S transition up to G2, and absent in θ-categories 1 (G1) and 6 (G2/M+G1) (except for one gene in the U2OS data). By contrast, numerous non-E2F targets were found in θ-categories 1 (G1) and 6 (G2/M+G1) (Fig 4C and 4D). This difference was statistically significant (p<0.001) and likely reflects G2/M-G1-specific transcriptional control, resulting in temporal compartmentalization and separation of E2F- and non-E2F-dependent transcription.

We further investigated the protein expression of a set of TFs to determine if their oscillatory mRNA behaviour was maintained after translation. Protein expression was examined by immunostaining, with the cell cycle phase determined by Fucci reporters, and by high-throughput image analysis of individual cells. This analysis showed that corresponding protein levels peaked in the same phase or one or two phases later than the mRNA. For example, the mRNA and protein expression of MYBL1 and TGIF1 peaked in the same phase (Fig 4E and 4F), whereas the mRNA level of HMGB2 peaked in G2/M, while its protein level peaked in G1 (Fig 4G). Likewise, the mRNA expression of *KDM5B* peaked in G2/M, but its protein level was highest in the G1 and S phases (Fig 4H). These results indicate that there is variability in translational control, which is nevertheless maintained in accordance with cell cycle oscillation.

Mapping our data onto known hierarchical relationships of TFs verified that the temporal component of such relationships could be readily identified with TriComp. For example (Fig 4I), the demethylase KDM5B, an upstream activator of E2F1 expression [25], peaked in G1 at the mRNA level and in G1 and S phases at the protein level, similar to the mRNA expression of its target gene E2F1 (Fig 3E). Likewise, the mRNA expression of TFs known to be E2F targets, such as MYBL2 [26] and HMGB2 [27], peaked in S and G2/M phases (Fig 4E and 4G). In summary, oscillations in TF mRNAs may be transformed into oscillations in protein expression and further into the expression of downstream target genes. Therefore, our TriComp analyses can be used as a starting point to construct networks of temporal causality.

### Oscillating developmental TFs assemble into transcriptional networks outside of S phase

**Fig 5:**
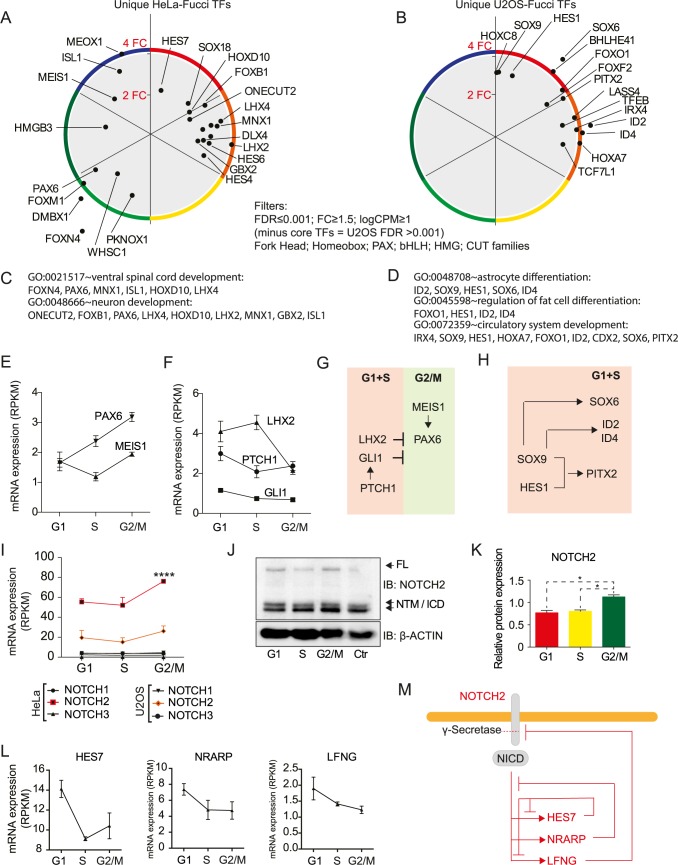
Transcriptional networks in G2/M-G1 phases are associated with developmental programmes. (A, B) Circular plot of θ and r coordinates for selected cell type-specific TF subfamilies expressed in (A) Hela-Fucci cells or (B) U2OS-Fucci cells (FDR ≤0.001, logCPM ≥1, FC ≥1.5). (C, D) GO enrichment analysis of the identified oscillating TFs against a background of all TFs. (E) NOTCH mRNA expression levels (RPKM) in HeLa-Fucci and U2OS-Fucci cells. (F) Representative western blot of total full-length (FL) NOTCH2 and the transmembrane/intracellular region (NTM/ICD) in sorted HeLa-Fucci cells. (G) Quantitation of western blot data for total NOTCH2 protein expression relative to the unsorted sample (Student’s t-test; * p<0.05). (H, J, K) mRNA expression of Notch signalling target genes HES7, NRARP and LFNG in the HeLa-Fucci data set (RPKM). (I) Map of a Notch-dependent oscillator as expressed during embryonic somitogenesis, indicating the key oscillators HES7, NRARP and LFNG. (L-M) Examples of known relationships between TFs identified to oscillate in synchrony with the cell cycle in the (L) HeLa-Fucci and (M) U2OS-Fucci data sets. Error bars denote SEM.

The analysis of TF distribution in Fig 3C and 3D showed that development-associated TF families such as Homeobox, Forkhead, HMG and zf-Z2H2 had varying patterns different from those of, e.g., the E2F and MYB families (Fig 3C and 3D). Oscillating TFs shared between HeLa and U2OS cells included SOX4, GLI1 and FOXO6 (peaking in G1) and KLF9, KDM5B and PRDM1 (peaking in G2/M-G1) (Fig 3E). These TFs are associated not only with development but also with cancer [28–36]. This observation prompted us to analyse TFs that are known to regulate development, focusing on TFs that were specific to either HeLa or U2OS cells. To this end, individual TFs from a number of TF families that control cell identity and morphogenesis (Forkhead; Homeobox; PAX; bHLH; HMG; CUT) were visualized as polar coordinates (FDR≤0.001, logCPM>1, FC≥1.5), showing a preference for θ-categories 1 (G1) and 2 (G1+S) and a complete absence from θ-category 3 (S) (Fig 5A and 5B). GO enrichment analysis (S5 Table) identified enrichment of neurodevelopmental TFs in HeLa cells (a human cervical carcinoma cell line [37]) (Fig 5C) and mesodermal TFs in U2OS cells (Fig 5D), reflecting the osteosarcoma origin of U2OS [38].

Candidate oscillating developmental programmes were next analysed in more detail by superimposing known hierarchical relationships of the identified TFs. In the HeLa transcriptome, PAX6, a regulator of neurodevelopment, was one of the TFs with the highest FC during the cell cycle, with a peak in G2/M (Fig 5A), which was also confirmed at the protein level (S4 Fig). A known activating TF upstream of PAX6, MEIS1 [39], had an expression profile similar to PAX6 (Fig 5E). Expanding the putative network around PAX6, the mRNA expression of the PAX6 repressor LHX2 peaked in G1+S, i.e., in an anti-phase manner with PAX6 (Fig 5F), reflecting its repressor function. Other oscillating repressors of PAX6 were identified in SHH signalling [40, 41]; both PTCH1 and GLI1 exhibited the highest mRNA expression in G1 phase in HeLa cells (Fig 5F). These data support the idea that hierarchical temporal relationships may be identified and assembled into networks (Fig 5G).

In U2OS cells, cell type-specific TFs synchronized with the cell cycle peaked in G1 (θ-category 1) and G1+S (θ-category 2) (Fig 5B). To map potential network connections, these TFs were overlaid with information on well-established interactions. This analysis revealed connections between SOX6, SOX9, PITX2 and ID2/ID4 (Fig 5H), supporting the association between the U2OS transcriptome and, for example, cardiac development [42, 43], as suggested by the GO associations (Fig 5D).

In conclusion, mapping cell-cycle-synchronized expression by TriComp in our data sets revealed networks of transcriptional regulators that involved more gene categories than has been generally believed.

### Cell-cell signalling via Notch peaks in G2/M-G1

Interestingly, PAX6 and FOXN4, which were found to oscillate over the cell cycle in HeLa cells and peaked in S+G2/M (θ-category 4) (Fig 5A), upregulate Notch signalling [44, 45]. FOXN4 directly controls DLL4, which peaked in G1 (θ-category 1) (S2 Table). Furthermore, Notch signalling targets were found to oscillate in our data, with HES7 and HES1 peaking in G1 phase in HeLa and U2OS cells, respectively (Fig 5A and 5B). Plotting the mRNA expression of NOTCH1-3 over the cell cycle confirmed that NOTCH2 expression peaked in G2/M in HeLa cells (θ-category 5), with a similar trend observed in the U2OS data set (Fig 5I). The less expressed NOTCH3 significantly oscillated and peaked in G1 in both HeLa and U2OS cells (S2 Table). The peak expression of full-length NOTCH2 in G2/M was also verified at the protein level in sorted HeLa-Fucci cells by western blotting using two different antibodies (Fig 5J and 5K, Panel A in S5 Fig). Analysing the potential oscillation of Notch target genes showed that NRARP and LFNG peaked in G1, similar to HES7 (Fig 5L). These genes act as a negative feedback loop to repress Notch gene expression and constitute a transcriptional circuit that plays a fundamental role in development during, for example, somitogenesis [46–49]. When exploring our data for receptors other than Notch, members of several classes of receptors were found to oscillate in HeLa and U2OS cells (Panel B in S4 Fig). Interestingly, among these were additional components of the somitogenesis network, such as WNT (FZD1/4/7/8/9) and FGF (FGFR3) signalling [46] and SHH (GLI1, PTCH, SMO) signalling [50] in HeLa cells (S5 Fig). Thus, visualizations based on TriComp analysis in combination with the superimposition of TF networks revealed that TF networks synchronize with the cell cycle, reproducing the hierarchy associated with development.

### The circadian molecular clock is synchronized with the cell cycle

The Notch and WNT pathways are known to be regulated by the circadian clock [51, 52], PAX6 is a direct target of the circadian protein CLOCK [53], and SOX4 is controlled by RORα, which is part of the circadian clock [54] (Fig 3E). The findings imply a coupling between the circadian clock and the cell cycle. Indeed, the circadian clock has previously been found to synchronize with the cell cycle in U2OS cells [15] and NIH3T3 cells [14]. Therefore, we examined if circadian clock constituents also oscillated in our data sets.

**Fig 6:**
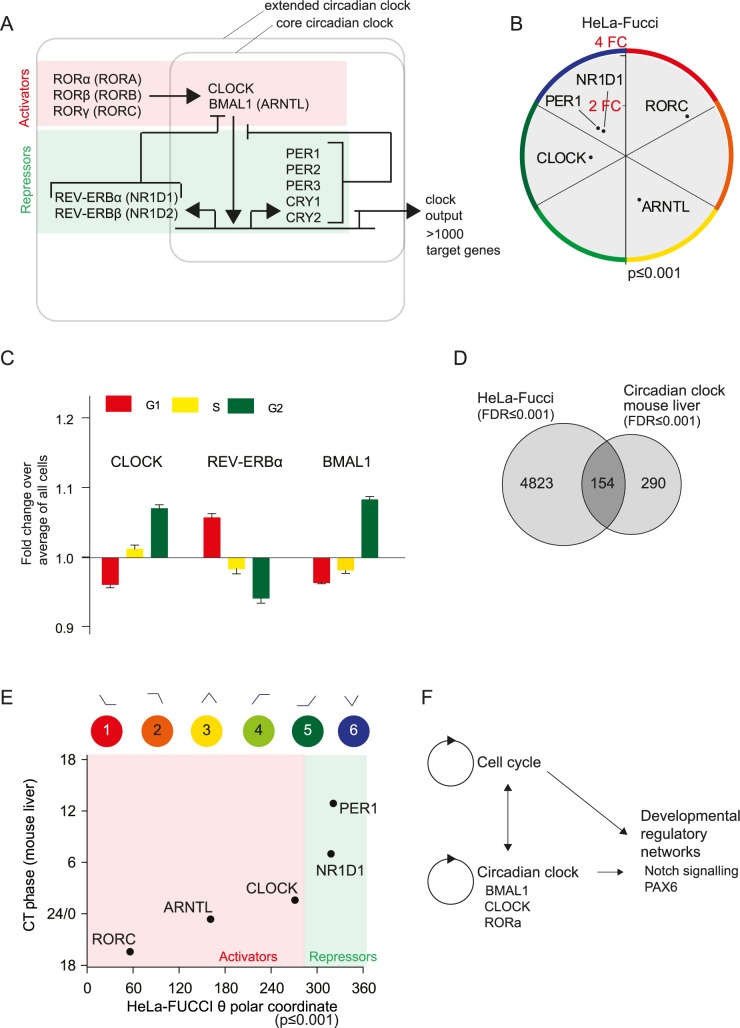
The circadian clock transcriptional network is synchronized with the cell cycle. (A) Schematic illustrating the feedback loops of the circadian clock oscillators. (B) Plot of the θ-value for core circadian genes in HeLa-Fucci cells (p-value≤0.001). (C) Protein expression levels of core components of the circadian clock in HeLa-Fucci cells analysed by correlating fluorescent immunostaining intensity to cell cycle phase determined by Fucci reporters or DNA content (DAPI); bars represent the mean of the logarithmic intensity of cells relative the average mean logarithmic intensity of all cells. Error bars denote SEM. (D) Comparison between cell cycle oscillating transcripts in HeLa-Fucci cells (FDR≤0.001) and a published circadian clock transcriptome in non-proliferating liver cells[55]. (E) Plot of the θ-values for core circadian genes in HeLa-Fucci cells (FDR≤0.001) versus the circadian peak values in liver cells reported by Yoshitane H, *et al*. 2014. (F) Proposed model of the integration of cell cycle, circadian clock and genes associated with development found to be synchronized with the cell cycle.

The intrinsic cellular circadian molecular clock consists of a transcriptional feedback loop, in which the BMAL1 (encoded by *ARNTL*) and CLOCK genes represent the main activating component and control the feedback repressors CRY1-2 and PER1-3 (overviewed in Fig 6A). Additional repressors are REV-ERBα and REV-ERBβ (encoded by *NR1D1* and *NR1D2*), and additional activators are RORα, RORβ and RORγ (encoded by RORA, RORB and RORC). In HeLa cells, genes from both the activating and repressing sides of the loop were synchronized with the cell cycle, but with a phase shift (Fig 6B). In U2OS cells, only the repressors were cell-cycle-synchronized (Panel A in S6 Fig).

We analysed the protein expression of BMAL1, CLOCK and REV-ERBα during the cell cycle by immunostaining of HeLa-Fucci cells and high-throughput image analysis using Fucci reporters (CLOCK and REV-ERBα) or DNA content (BMAL1) as indicators of the cell cycle phase. The protein levels of CLOCK and BMAL1 were cell-cycle-synchronized, peaking in G2/M, while REV-ERBα peaked at G1 (Fig 6C). Our observations thus support previous reports of synchronization between the cell cycle and the circadian clock [14, 15].

To extend the analyses to genes downstream of the circadian clock, the transcriptomes of HeLa and U2OS cells were compared to a published mouse liver circadian transcriptome data set [55]. Importantly, the liver data set provides information regarding circadian oscillations in predominantly non-proliferative cells, thus reducing the mis-attribution of cell cycle genes to the circadian network. A Venn analysis showed that 35% of the circadian-controlled genes in the liver significantly oscillated in HeLa cells (Fig 6D). Plotting the θ-values of our HeLa data set against the circadian clock variable Circadian Time (CT) for each gene in the circadian transcriptional feedback loop from Yoshitane *et al*. 2004, verified the temporal segregation of the activating and repressing parts of the molecular clock loop (Fig 6E). Interestingly, a number of TFs regulating development were identified to be circadian oscillating in the Yoshitane *et al*. data, suggesting that the circadian clock contributes to synchronize developmental transcriptional regulators with the cell cycle (Fig 6F).

To examine the association between the cell cycle and circadian transcriptomes in proliferating cells, we compared the HeLa and U2OS data sets to a transcriptome of oscillating clock genes in NIH/3T3 cells [56]. Fifty percent of the circadian-oscillating genes varied over the cell cycle in HeLa cells, and 13% in U2OS cells (Panel B in S6 Fig). Notably, 27 transcripts were shared between the circadian clock and the cell cycle in both HeLa and U2OS cells, including E2F8, Cyclin B2 (CCNB2) and CDK1 (Panel B in S6 Fig), likely reflecting synchronization between the circadian clock and the cell cycle.

In conclusion, our data suggest that the circadian clock might be an intermediary step in the synchronization between the cell cycle and the networks of TFs associated with development, oscillating in synchrony with the cell cycle (Fig 6F).

## Discussion

In this study, we identified that an unexpectedly large number of developmentally associated TFs assemble into networks that are synchronized with the cell cycle in cancer cells. This results in a temporal compartmentalization, analogous to spatial patterning during embryogenesis. The circadian clock was similarly found to be synchronized with the cell cycle, indicating a link between the cell cycle and some of the identified TF networks.

The identification of TF networks was greatly aided by a novel algorithm, TriComp, that allows for easy visualization of expression patterns over the cell cycle, providing more information than mere identification of a single point of peak expression. In addition, the algorithm enables comparisons of data sets even in cases where the underlying temporal variables are calculated differently.

Reactivation of developmental genes has attracted increased interest in the cancer field [57–59], particularly in cancer stem cells. Examples of TFs that control morphogenesis and play a role in cancer that were identified to oscillate in our data include PAX6 [60] and GLI1 [41]. Notably, PAX6 was previously found to be dynamically expressed during the cell cycle in neural progenitor cells, affecting proliferation as well as fate choice during differentiation [61]. In our study, knowledge from TF networks controlling embryo development was used to decipher relationships between TFs that might assemble into similar networks over the cell cycle. Our analyses supported the idea that cell-cycle-synchronized expression of developmental genes might be a consequence of feed-forward transcriptional activation or repression by “non-core” TFs.

In addition to transcriptional feed-forward coordination, synchronized transcription may also be a consequence of cell cycle-governed cell signalling. If cell surface receptors are differentially expressed during the cell cycle, the influence of external factors may occur in a cell cycle-dependent manner, suggesting another dynamic dimension of regulation. Such phase dependent signalling has been established for the WNT/Frizzled/β-catenin pathway [8, 9]. An example of temporally restricted signalling in our data was Notch signalling, which is a central factor in development, controlling cell identity and proliferation [62]. Activated NOTCH2-ICD protein expression peaked in G2/M and was followed by expression of Notch target genes in G1 in HeLa cells. In somitogenesis, Notch interacts with the WNT/Frizzled and FGFR signalling pathways (reviewed in [46, 63, 64]) through a critical negative feedback loop mediated by HES1 and HES7 [49, 65]. This feedback function appeared to be present in the HeLa and U2OS cell cycles as well, as HES1 and HES7 oscillated in our data and peaked in G1, where NOTCH2 expression itself was downregulated.

One unresolved question is what factor(s) synchronize “non-core” transcription and signalling with the cell cycle. A candidate is the circadian clock. Our data show that the circadian clock oscillates in synchrony with the cell cycle in HeLa cells, as was previously shown for U2OS [15] and NIH/3T3 [56] cells. Signalling via the Notch and the WNT/Frizzled pathways has previously been shown to be controlled by the circadian clock [51, 52], and several of the TFs identified in our data (e.g., PAX6) may be targets of the circadian clock [53]. Further supporting such a conclusion is that the circadian TFs CLOCK and BMAL1 peaked in G2, i.e., the same phase as NOTCH2 in our data. However, the reciprocal interaction and synchronization between the cell cycle and the circadian clock, as well as other networks suggested by our data, pose a challenge when trying to identify which oscillatory system is in control of the expression profile of a particular gene.

Non-core cell cycle transcriptional networks such as the circadian clock resemble the non-essential transcription factor networks that oscillate in a self-sustained manner in yeast [12, 13]. One outcome from interactions between cellular oscillators is the establishment of temporally constrained signalling and gene expression, i.e., temporal compartmentalization. Such compartmentalization may provide opportunities for unique combinatorial effects when unique sets of oscillating TFs are temporally co-expressed. This resembles the processes whereby spatial compartmentalization and patterning is established in development [66, 67]. An additional interpretation of the data is that cell identity oscillates during the cell cycle, and this oscillation might determine the onset and direction of differentiation. Altogether, these perspectives reveal unexpected dynamics in transcriptional states, between which a cancer cell fluctuates when progressing through the cell cycle. Understanding these discrete states will have implications in target selection when developing cancer cell therapies, as expression levels as well as the cellular context of the target may change over the cell cycle. As the circadian rhythm appears to be a component in controlling the phase of these oscillations, our data suggest that the time of drug administration can be chosen to match the peak expression of the target, allowing for a higher therapeutic efficacy.

## Experimental procedures

### Cell culture

HeLa.S-Fucci and U2OS-Fucci are variants of the human cervical carcinoma cell line HeLa.S and the osteosarcoma cell line U2OS that express mKo2-hCdt1(30–120) and mAg-hGem(1–110)[20]. The HeLa.S-Fucci cell line was obtained from Riken Cell Bank, and the U2OS-Fucci cell line was a kind gift from Dr. Masai at the Tokyo Metropolitan Institute of Medical Science, Japan.

HeLa.S-Fucci cells were grown in Dulbecco's modified Eagle's medium (DMEM), and U2OS-Fucci cells were grown in McCoy’s modified medium, both supplemented with 10% foetal bovine serum (FBS), 5% Glutamax and 100 U/ml penicillin-streptomycin (all from Gibco, Invitrogen), at 37°C in a 5% CO_2_ humidified atmosphere. The cells were dissociated by trypsinization (TrypLE Express 1x) (Gibco, Invitrogen).

### Flow cytometry

Cells were grown to ~80% confluency, collected by trypsinization, washed with complete DMEM/McCoy’s with 10% FBS, 5% Glutamax and 100U/ml penicillin-streptomycin, spun down by centrifugation at 200 g for 5 minutes and resuspended in culture medium. The cells were sorted based on different laser emission wavelengths simultaneously by defining three regions for sorting: G1, S and G2/M phases. Additionally, a control group of all three phases were collected. See S1 Table for Minimal Information of a Flow Cytometry experiment (MIFlowCyt).

For western blot 500 000 cells per phase were collected in PBS, spun down and resuspended in 100 μl of lysis buffer (1% SDS, 10% glycerol, 100 mM Tris/HCl pH 7.5). For each phase, post-sorting control was performed (see Panel B in S2 Fig).

### RNA extraction, RNA sequencing and data analysis

Total RNA was extracted from sorted cells using an RNeasy Micro Kit (Qiagen) following the manufacturer’s instructions. The RNA concentration and quality were assessed using the Qubit RNA assay kit (Invitrogen). We used 300 ng of total RNA to prepare the TruSeq library, for which we used the Illumina Low-Throughput TruSeq RNA Sample Preparation Kit protocol, resulting in barcoded cDNA. Next, 50 ng of barcoded TruSeq products were used for Illumina RNA sequencing on an Illumina HiSeq 2000 sequencer to generate single-end 50 or 51-nucleotide reads according to the manufacturer’s protocol and as previously described [68]. The expression levels of each sample were normalized as Reads Per Kilobase Per Million (RPKM) by dividing the read count of each transcript model with its length and scaling the total per sample to one million.

### Transcriptome data analysis

Differential gene expression analysis was performed on the mapped read counts using an ANOVA-like approach in the edgeR package in R [23], correcting for common, trended and tagwise disparity. For the HeLa cells (three batches), the GLM model was simply (Expression ~ Cell cycle phase). For the U2OS cells (two batches), the samples were corrected for batch-specific effects by including the batch in the GLM model (Expression ~ Cell cycle phase + Batch). This analysis supplied p-values, logFC differences between groups, and FDR-values.

A “Highest-logFC” variable was created by finding the largest logFC difference between any two cell cycle phases.

TriComp: Converting logarithmic FCs into polar relationship descriptor variables

Starting with the log2FC differences between groups 2 and 1, “logFCGr2overGr1”, and groups 3 and 1, “logFCGr3overGr1”, the TriComp algorithm consists of four equations. First, it creates two variables (*a* and *b*) used for plotting the circular graphs, in which direction from the centre denotes the relationship between the three groups and distance from the centre denotes the intensity of the respective relationship:

$$a=logFCGr3overGr1+Sin\;\left(\frac{Pi}{6}\right)*logFCGr2overGr1$$ (1)

$$b=Cos\;\left(\frac{\pi }{6}\right)*logFCGr2OverGr1$$ (2)

Then, TriComp calculates the polar coordinates (*r*, θ) with (*a* = 0, *b* = 0) used for (*r* = 0) and θ starting at 0 straight up and going clockwise. Calculating θ requires four different equations depending on what quadrant it belongs to.

$$r=\sqrt{a^{2}+b^{2}}$$ (3)

$$\begin{array}{l} IF\;\left(a\geq 0\;\&\;b>0\right)\;THEN\;\theta =tan^{-1}\;\left(\frac{a}{b}\right)\;ELSEIF\;\left(a\geq 0\;\&\;b<0\right)\;THEN\;\theta =180{}^{\circ}-\\ tan^{-1}\;\left(\frac{a}{b}\right)\;ELSEIF\;\left(a<0\;\&\;b<0\right)\;THEN\;\theta =180{}^{\circ}+tan^{-1}\;\left(\frac{a}{b}\right)\;ELSEIF\;(a<0\;\&\;b>\\ 0)\;THEN\;\theta =360{}^{\circ}-tan^{-1}\;\left(\frac{a}{b}\right)\;ELSEIF\;\left(b=0\;\&\;a>0\right)\;THEN\;\theta =90{}^{\circ}\;ELSEIF\;(b=\\ 0\;\&\;a<0)\;THEN\;\theta =270{}^{\circ} \end{array}$$ (4)

Calculating which group each gene belongs to according to Fig 2A is performed using the following equation:

$$Group=Int\;\left(\frac{\theta }{60{}^{\circ}}\right)+1$$ (5)

In the graph using the Euclidean coordinates *a* and *b*, θ and *r* are the corresponding polar coordinates.

### Immunofluorescence

For antibodies generated within the Human Protein Atlas (MYBL2, TGIF1, HMGB2, KDM5B), immunostaining was performed on cells grown on glass bottom plates (Whatman Inc.) coated with 50 μg of 12.5 μg/ml human fibronectin (VWR). Approximately 8 000 cells were seeded in each well and incubated at 37°C for 24 hours. After washing with PBS, the cells were fixed with 40 μl of 4% ice cold PFA (Sigma Aldrich) dissolved in growth medium supplemented with 10% serum for 15 minutes and permeabilized with 40 μl of 0.1% Triton X-100 (Sigma Aldrich) in PBS for 3x5 minutes. Primary antibodies (HPA055416, MYBL2; HPA062160, TGIF1; HPA053314, HMGB2; HPA053723, KDM5B) were diluted to 2.5 μg/ml in blocking buffer (PBS + 4% FBS) containing 3.3 μg/ml mouse anti-tubulin (Abcam, ab7291, Cambridge, UK). After washing with PBS, diluted primary antibodies were added (40 μl/well), and the plates were incubated overnight (ON) at 4°C. After the ON incubation, all wells were washed with PBS for 3x10 minutes. Goat anti-mouse Alexa Fluor 405-conjugated (A31553, ThermoFisher) and goat anti-rabbit Alexa Fluor 647-conjugated (A21245, ThermoFisher) secondary antibodies diluted to 2.5 μg/ml in blocking buffer were added, and the plates were incubated for 90 minutes at room temperature (RT). After washing with PBS, all wells were mounted with PBS containing 76.5% glycerol. Image acquisition was performed with a Leica SP5 confocal microscope equipped with a 63-x/1.4 NA oil immersion objective with the support of LAS AF matrix software. Six images were acquired per sample at RT in three sequential steps with the following scanning settings: pinhole 1 Airy unit, 16-bit acquisition and a pixel size of 80x80 nm. The z focus-level was manually adjusted to represent the best visualization of the target protein. The detector gain remained constant for the Fucci markers and microtubules across all samples.

For immunostaining of circadian clock proteins and PAX6, cells were seeded in 96-well glass bottom microplates (BD Falcon) at a density 2.5x10^3^ cells/well ON. The next day, the cells were fixed in 4% paraformaldehyde (PFA) in PBS for 15 minutes at RT. Fixed cells were washed with PBS and permeabilized with 0.3% Triton X-100 in PBS (PBST). After blocking (1% BSA in PBST) for 1 hour at RT, the cells were incubated with a primary antibody diluted in 1% BSA in PBST ON at 4°C. On day 3, the cells were washed 3X with PBST. Before incubation with a fluorescence-labelled secondary antibody, the cells were blocked with 1% BSA in PBST for 30 minutes at RT. To remove cellular RNA, cells were incubated with RNase (20 μg/ml) in PBS for 1 hour at 4°C. Nuclei were counterstained with DAPI (5 μg/ml) in staining buffer (100 mM Tris, pH 7.8; 150 mM NaCl; 1 mM KCl; 0.5 mM MgCl_2_; 0.1% Nonidet P-40) for 30 minutes at RT.

The primary antibodies used were as follows: Pax6 (1:300; Biolegend), BMAL1 (1:100; NB100-2288, Novus Biological), CLOCK (1:100; PA1-520, Pierce), and REV-ERBα (1:200; H00009572-M02, Abnova). The secondary antibody used was an Alexa Fluor 647-conjugated anti-rabbit antibody (1:1000, Invitrogen).

For EdU incorporation analysis, the cells were grown, fixed and permeabilized as above, but incubated with 10μM EdU 30 min before fixation, then after permeabilization incubated 30min at room temperature with 2μM Alexa 647-conjugated Azide, 10 mM ascorbic acid, 2.5 mM CuSo_4_ in PBS. DAPI staining was performed by a 1h incubation at 4°C with10μg/mL RNAse A followed by 10 minutes of 1 μg/mL DAPI in PBS at room temperature.

All plates were imaged on an ImageXpress imaging system (Molecular Devices) at 10X magnification, and segmentation of nuclei and measurement of fluorescence intensities were performed in CellProfiler [69]. Per-object measurements were categorized and intensities were analysed in R.

### Western blot

Protein lysates of sorted cells were separated by SDS-PAGE on 8% acrylamide gels and blotted onto PVDF membranes (Millipore). The membranes were incubated with primary rabbit anti-NOTCH2 antibodies N4913 (Sigma Aldrich) (Fig 6F and 6G) or D67C8 (Cell Signaling Technology) (Fig 6G, Panel A in S4 Fig) or a mouse anti-β-ACTIN (Abcam, ab8226) antibody ON at 4°C and with HRP-conjugated secondary antibodies (1:1000 Sigma Aldrich) at RT for 1 h. The membranes were developed using an ECL detection reagent (GE Healthcare) and a charge-coupled device camera (Bio-Rad, US). Non-saturated immunoblots were analysed by ImageJ, and protein abundances were normalized to β-ACTIN.

### Statistical tools, visualization tools and bioinformatics

R[70] with attached packages ggplot2 [71], dplyr [72], mapplots [73] and XNomial [74] was used for statistical analysis, processing of immunofluorescence data, and visualization. GraphPad PRISM 6 was used for statistical analyses and data visualization. DAVID [75] (https://david.ncifcrf.gov) was used for gene ontology data analyses.

### Data availability

The raw read counts, RPKM values and statistical data from EdgeR have been made available as a GEO submission (#GSE104736)

## Supporting information

S1 Fig Non-synchronized Fucci based cell phase sorting and RNA sequencing to identify cycling transcripts. (A) Using high-throughput imaging analysis, U2OS Fucci cells were stained and imaged for DAPI, EdU incorporation and Fucci status. Results are first shown separately (top and bottom left), then classified according to DNA status according to DAPI and EdU, and summaries overlaid on a grid of the Fucci variables (right).
(EPS)

S2 Fig Examples of relative distribution of transcripts in cell cycle phases. (A) Live HeLa-Fucci cells were sorted in 3 cell cycle populations based on expression of Fucci markers. (B) Post-sort validation was done by flow cytometry analysis of expression of Fucci markers as well as of DNA content (staining by propidium iodide, PI). (C, D) Analysis of transcriptome data from HeLa-Fucci and U2OS-Fucci cells by plotting the maximum fold-change (FC) difference between any two cell cycle phase groups against the logarithmic expression level (logarithmic Counts per Million reads (logCPM)) for each transcript. The vast majority of all genes with FDR≤0.001 (data indicated in red) also had an FC of at least 1.1. (E, F) Summary of statistical analysis of oscillating transcripts in (E) HeLa-Fucci and (F) U2OS-Fucci cells. (G) A table showing example *θ* values, their categories and relative gene expression profiles between the three cell cycle phases.
(TIF)

S3 Fig (A) A comparison between HeLa-Fucci cell cycle transcriptome and the Whitfield et al. data set [19] indicates number of shared transcripts. (B) Distribution plots of the *θ* value for HeLa-Fucci versus the full hit-list of the Seed Match Category reported by [18]. (C) STRING analysis (using the web interphase available at http://string-db.org) of TFs synchronized with the cell cycle at FDR≤0.001. The STRING analysis was set at highest confidence (0.900) and included all interaction sources.
(EPS)

S4 Fig (A) Protein expression levels of PAX6 in HeLa-Fucci cells analyzed by fluorescent imaging correlating immunostaining of PAX6 to cell cycle phase determined by DNA content (DAPI), represented as boxplots. (B) Examples of receptors and associated proteins significantly oscillating in HeLa and U2OS cells at FDR≤0.001.
(EPS)

S5 Fig A schematic illustration of a network incorporating FGF, Notch and WNT signaling oscillates over the cell cycle. (EPS)

S6 Fig Molecular clock synchronization with the cell cycle. (A) Plot of the θ-value for core circadian genes in U2OS-Fucci cells (p-value≤0.001). (B) Venn diagram between cell cycle oscillating transcripts in U2OS-Fucci (FDR≤0.001), HeLa-Fucci cells (FDR≤0.001) and published circadian clock transcriptome in non-proliferating liver cells [55].
(EPS)

S1 Table MiFlowCyt—Hela Fucci and U2OS Fucci sortings. (PDF)

S2 Table RNA sequencing and TriComp data. (CSV)

S3 Table GO cell cycle term summaries. (XLSX)

S4 Table Transcription factor results. (XLSX)

S5 Table GO term enrichment of developmental transcription factors. (XLSX)
