# Supplementary figures and images for: Comparative cell cycle transcriptomics reveals synchronization of developmental transcription factor networks in cancer cells
Source: PLoS One. 2017 Dec 11;12(12):e0188772. doi: 10.1371/journal.pone.0188772 (PMC5724894; doi:10.1371/journal.pone.0188772)

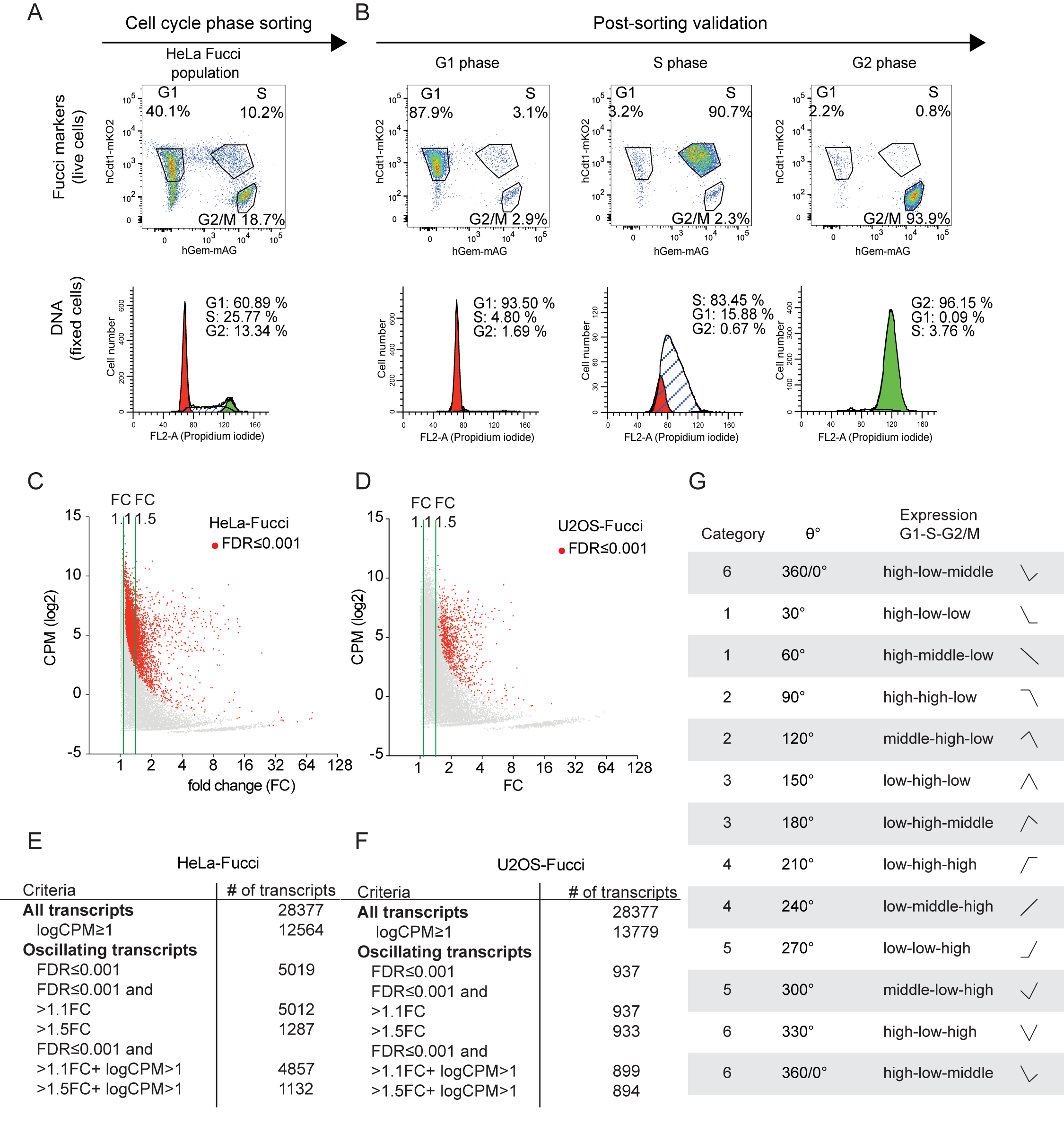

Supplement: S2 Fig — (A) Live HeLa-Fucci cells were sorted in 3 cell cycle populations based on expression of Fucci markers. (B) Post-sort validation was done by flow cytometry analysis of expression of Fucci markers as well as of DNA content (staining by propidium iodide, PI). (C, D) Analysis of transcriptome data from HeLa-Fucci and U2OS-Fucci cells by plotting the maximum fold-change (FC) difference between any two cell cycle phase groups against the logarithmic expression level (logarithmic Counts per Million reads (logCPM)) for each transcript. The vast majority of all genes with FDR≤0.001 (data indicated in red) also had an FC of at least 1.1. (E, F) Summary of statistical analysis of oscillating transcripts in (E) HeLa-Fucci and (F) U2OS-Fucci cells. (G) A table showing example θ values, their categories and relative gene expression profiles between the three cell cycle phases. (TIF) [file pone.0188772.s002.tif]
