# Supplementary material for: Comparative cell cycle transcriptomics reveals synchronization of developmental transcription factor networks in cancer cells
Source: PLoS One. 2017 Dec 11;12(12):e0188772. doi: 10.1371/journal.pone.0188772 (PMC5724894; doi:10.1371/journal.pone.0188772)
Supplement: S1 Table — (PDF) [file pone.0188772.s007.pdf]

Supplementary table S1a: Minimum Information about a Flow Cytometry Experiment (MIFlowCyt)

**Requirement**	**Please Include Requested Information**
1.1. Purpose	Sort 200 000 cells per phase for RNA sequencing and 500 000 cells for immunoblotting from HeLa S. FUCCI cell line.
1.2. Keywords	Cell cycle, circadian molecular clock, FUCCI
1.3. Experiment variables	Cell cycle-sorted HeLa cell lines using FUCCI reporters.
1.4. Organization name and address	Department of Cytokinetics, Institute of Biophysics CAS, v.v.i., Královopolská 135, 612 65 Brno, Czech Republic
1.5. Primary contact name and email address	Michael Andäng, michael.andang@ki.se,<br><br>Department of Physiology and Pharmacology, Karolinska Institutet, 171 77 Stockholm, Sweden.<br><br>Central European Institute of Technology, Masaryk University, 625 00 Brno, Czech Republic.
1.6. Date or time period of experiment	18.6.2010 – 20.12.2012
1.7. Conclusions	200 000 cells per phase were sorted for subsequent analyses into 200 µl of RLT lysis buffer with 1% 2-mercaptoethanol for the RNA sequencing, and 500 000 cells per phase for immunoblotting into 100 µl of lysis buffer.
1.8. Quality control measures	Cell aggregates and debris were excluded from the analysis based on FCS-A vs. FCS-H, and FSC-A vs. SSC-A a dual-parameter dot plots. For each phase post-sorting control was done.
2.1.1.1. (2.1.2.1., 2.1.3.1.) Sample description	Cells were grown to sub-confluency, collected by trypsinisation, washed with full DMEM/McCoy's with 10% serum, 5% Glutamax and 100U/ml penicillin-streptomycin, spinned down by centrifugation at 200 g for 5 minutes and resuspended in culture media before sorting.
2.1.1.2. Biological sample source description	HeLa.S FUCCI human cervical carcinoma cell line – source: Riken Cell Bank.
2.1.1.3. Biological sample source organism description	Human.
2.1.2.2. Environmental sample location	-
2.3. Sample treatment description	Samples were not treated.

2.4. Fluorescence reagent(s) description

Marker	Laser	Manufacturer	Power	Detector	Dichroic Mirror // Band Pass Filter (nm)	Instalation date	Instrument
mAG-hGem	488	Coherent Sapphire	100 mW	F - Blue Octagon	505LP // 525/50	2009	FACSAria II Sorp
mKO2-hCdt1	488	Coherent Sapphire	100 mW	E - Blue Octagon	550LP // 575/25	2009	FACSAria II Sorp

3.1. Instrument manufacturer

BD Biosciences, San Jose, CA 95131 USA

3.2. Instrument model

BD FACSAria™ II Cell Sorter (ver. Sorp 4L), Serial number: PY500001

3.3. Instrument configuration and settings

488nm laser excitation:

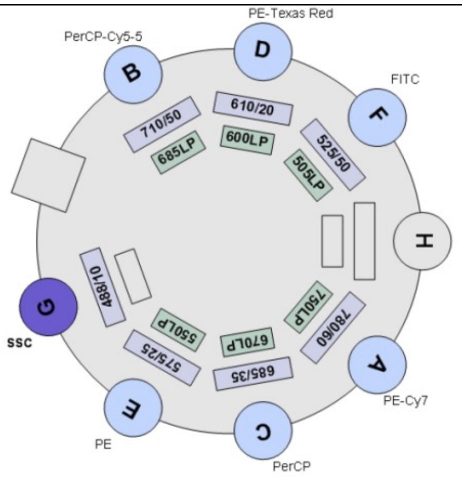

4.1. List-mode data files

FCS data files can be obtained by contacting Michael Andäng after this work has been published.

4.2. Compensation description

Manual compensation was applied based on quantification of medians of positive and negative populations on both markers hGem-mAG (detector F) vs. hCdt1-mKO2 (detector E).

4.3. Data transformation details

FACSDiva (Version 6.1.3; BD Biosciences) and FlowJo software (Version 10.0.7, Tree Star, Ashland, OR, USA) was used for data visualization.

4.4.1. Gate description

Single cells: FSC-A vs. FSC-H gate to define single cells from doublets.
Debris exclusion: FSC-A vs. SSC-A gate to define compact population of cells without debris.
G1: hGem-mAG (detector F) vs. hCdt1-mKO2 (detector E) gate to define G1 cell cycle phase.
S: hGem-mAG (detector F) vs. hCdt1-mKO2 (detector E) gate to define G1/S cell cycle phase
G2: hGem-mAG (detector F) vs. hCdt1-mKO2 (detector E) gate to define S/G2/M cell cycle phase

4.4.2. Gate statistics

Please see the next section.

4.4.3. Gate boundaries

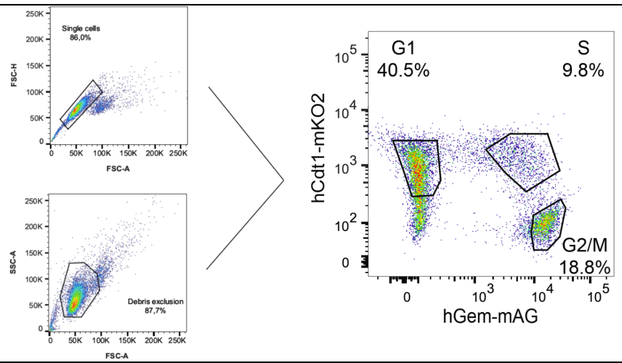

Supplementary table S1b: Minimum Information about a Flow Cytometry Experiment (MIFlowCyt)

Requirement	Please Include Requested Information
1.1. Purpose	Sort 200 000 cells per phase for RNA sequencing of U2OS FUCCI cell line.
1.2. Keywords	Cell cycle, FUCCI
1.3. Experiment variables	Cell cycle-sorted U2OS cell line using FUCCI reporters.
1.4. Organization name and address	Karolinska Institutet CMM<br><br>Flow Cytometry facility L8:03, 17176 Stockholm, Sweden
1.5. Primary contact name and email address	Mikael Altun, Mikael.altun@ki.se<br><br>Department of Medical Biochemistry and Biophysics, Karolinska Institutet, 171 77 Stockholm, Sweden.
1.6. Date or time period of experiment	7.10.2013 – 28.1.2014
1.7. Conclusions	200 000 cells per phase were sorted for subsequent analyses into 200 µl of Qiazol Lysis Reagent (Qiazol)
1.8. Quality control measures	Cell aggregates and debris were excluded from the analysis based on FSC-A vs SSC-A and FSC-A vs FSC-W
2.1.1.1. (2.1.2.1., 2.1.3.1.) Sample description	Cells were grown to sub-confluency, collected by trypsinisation, washed with full McCoy's with 10% serum, 5% Glutamax and 100U/ml penicillin-streptomycin, spun down by centrifugation at 500 g for 5 minutes and resuspended in culture media before sorting.
2.1.1.2. Biological sample source description	U2OS FUCCI human osteosarcoma cell line – source: Gift from Dr. Masai at the Tokyo Metropolitan Institute of Medical Science, Japan.
2.1.1.3. Biological sample source organism description	Human.
2.1.2.2. Environmental sample location	-
2.3. Sample treatment description	Samples were not treated.
2.4. Fluorescence reagent(s) description	(see table below)

Marker	Laser	Manufacturer	Power	Detector	Dichroic Mirror // Band Pass Filter (nm)	Instalation date	Instrument
mAG-hGem	488	Coherent	150mW	3	555DLP//530/40	01/2002	MoFlo legacy

<table>
<tr><td></td><td>mKO2-hCdt1</td><td>561</td><td>Melles Griot</td><td>35mW</td><td>6</td><td>605DLP//585/29</td><td>01/2002</td><td>MoFlo Legacy</td></tr>
<tr><td>3.1. Instrument manufacturer</td><td colspan="8">Beckman Coulter, USA</td></tr>
<tr><td>3.2. Instrument model</td><td colspan="8">MoFlo Legacy</td></tr>
<tr><td>3.3. Instrument configuration and settings</td><td colspan="8">488nm laser excitation: 530/40 (detector 3)
561nm laser excitation: 585/29 (detector 6)</td></tr>
<tr><td>4.1. List-mode data files</td><td colspan="8">-</td></tr>
<tr><td>4.2. Compensation description</td><td colspan="8">Manual compensation was applied based on quantification of medians of positive and negative populations on both markers hGem-mAG (detector 3) vs. hCdt1-mKO2 (detector 6).</td></tr>
<tr><td>4.3. Data transformation details</td><td colspan="8">Summit (V4.3.02) was used for data visualization.</td></tr>
<tr><td>4.4.1. Gate description</td><td colspan="8">Single cells: FSC-A vs. FSC-W gate to define single cells from doublets.
Debris exclusion: FSC-A vs. SSC-A gate to define compact population of cells without debris.
G1: hGem-mAG (detector 3) vs. hCdt1-mKO2 (detector 6) gate to define G1 cell cycle phase.
S: hGem-mAG (detector 3) vs. hCdt1-mKO2 (detector 6) gate to define G1/S cell cycle phase
G2: hGem-mAG (detector 3) vs. hCdt1-mKO2 (detector 6) gate to define S/G2/M cell cycle phase</td></tr>
<tr><td>4.4.2. Gate statistics</td><td colspan="8">Please see the next section.</td></tr>
<tr><td>4.4.3. Gate boundaries</td><td colspan="8">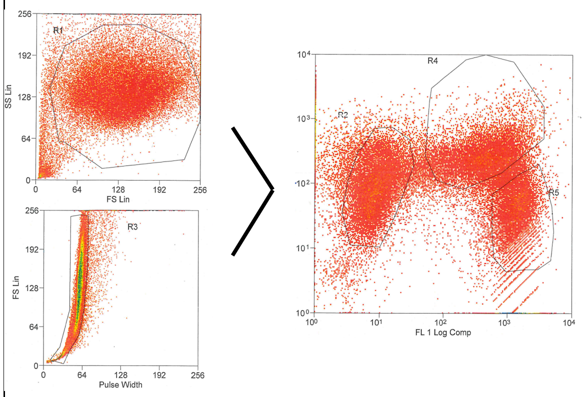
</td></tr>
</table>
